# Use of Nirsevimab for the Prevention of Respiratory Syncytial Virus Disease Among Infants and Young Children: Recommendations of the Advisory Committee on Immunization Practices — United States, 2023

**DOI:** 10.15585/mmwr.mm7234a4

**Published:** 2023-08-25

**Authors:** Jefferson M. Jones, Katherine E. Fleming-Dutra, Mila M. Prill, Lauren E. Roper, Oliver Brooks, Pablo J. Sánchez, Camille N. Kotton, Barbara E. Mahon, Sarah Meyer, Sarah S. Long, Meredith L. McMorrow

**Affiliations:** ^1^Coronavirus and Other Respiratory Viruses Division, National Center for Immunization and Respiratory Diseases, CDC; ^2^Watts Healthcare Corporation, Los Angeles California; ^3^Nationwide Children’s Hospital, The Ohio State University College of Medicine, Columbus, Ohio; ^4^Harvard Medical School, Boston, Massachusetts; ^5^Immunization Services Division, National Center for Immunization and Respiratory Diseases, CDC; ^6^Drexel University College of Medicine, Philadelphia, Pennsylvania.

**Summary**

**What is already known about this topic?**

In July 2023, the Food and Drug Administration approved nirsevimab, a long-acting monoclonal antibody, for prevention of respiratory syncytial virus (RSV) lower respiratory tract disease in infants.

**What is added by this report?**

On August 3, 2023, the Advisory Committee on Immunization Practices recommended nirsevimab for infants aged <8 months born during or entering their first RSV season and for infants and children aged 8–19 months who are at increased risk of severe RSV disease entering their second RSV season.

**What are the implications for public health practice?**

Nirsevimab can prevent severe RSV disease among infants and children aged <20 months at increased risk for severe RSV disease.

## Abstract

Respiratory syncytial virus (RSV) is the leading cause of hospitalization among U.S. infants. In July 2023, the Food and Drug Administration approved nirsevimab, a long-acting monoclonal antibody, for passive immunization to prevent RSV-associated lower respiratory tract infection among infants and young children. Since October 2021, the Advisory Committee on Immunization Practices (ACIP) Maternal and Pediatric RSV Work Group has reviewed evidence on the safety and efficacy of nirsevimab among infants and young children. On August 3, 2023, ACIP recommended nirsevimab for all infants aged <8 months who are born during or entering their first RSV season and for infants and children aged 8–19 months who are at increased risk for severe RSV disease and are entering their second RSV season. On the basis of pre–COVID-19 pandemic patterns, nirsevimab could be administered in most of the continental United States from October through the end of March. Nirsevimab can prevent severe RSV disease among infants and young children at increased risk for severe RSV disease.

## Introduction

In July 2023, the Food and Drug Administration (FDA) approved nirsevimab (Beyfortus, Sanofi and AstraZeneca), a long-acting monoclonal antibody, for the prevention of respiratory syncytial virus (RSV)–associated lower respiratory tract infection (LRTI) among infants and children aged <24 months (*1*).* Nirsevimab is administered as a 1-dose intramuscular injection shortly before or during the RSV season (typically fall through spring).^†^ Since October 2021, the Advisory Committee on Immunization Practices (ACIP) Maternal and Pediatric RSV Work Group (Work Group) has reviewed data on RSV among infants and young children and evidence regarding the safety and efficacy of nirsevimab, and assessed the quality of the efficacy and safety evidence using the Grading of Recommendations, Assessment, Development, and Evaluations (GRADE) framework (*2*,*3*). The Evidence to Recommendation (EtR) Framework was used to develop recommendations (*4*,*5*). Evidence regarding potential use of nirsevimab was presented to ACIP at meetings during June 2022–August 2023. On August 3, 2023, ACIP recommended nirsevimab for infants aged <8 months who are born during or entering their first RSV season and for infants and children aged 8–19 months who are at increased risk for severe RSV disease and are entering their second RSV season.

### RSV Among Infants and Young Children

RSV infection is the leading cause of hospitalization among U.S. infants (*6*); most children are infected during the first year of life, and nearly all have been infected by age 2 years (*7*,*8*). Infants with RSV infection frequently develop bronchiolitis, an LRTI that can be severe and result in hospitalization. Approximately 50,000–80,000 RSV-associated hospitalizations (*9*,*10*) and 100–300 RSV-associated deaths (*11*,*12*) occur annually among U.S. infants and children aged <5 years.

The rate of RSV-associated hospitalization among infants born at ≤30 weeks’ gestation (premature) is three times that of term infants (*13*). Premature infants also have higher rates of RSV-associated intensive care unit (ICU) admission (*14*). Although prematurity is a recognized risk factor for RSV-associated hospitalization, RSV is also the leading cause of hospitalization among healthy term infants. An estimated 79% of infants and children aged <2 years hospitalized with RSV have no underlying medical conditions (*13*).

Before licensure of nirsevimab, the only FDA-approved product to prevent severe RSV disease among infants and young children was palivizumab, another monoclonal antibody. However, the American Academy of Pediatrics (AAP) recommends palivizumab only for children with certain underlying medical conditions (comprising <5% of all infants), and its use is further limited by high cost and the requirement for monthly dosing (*15*,*16*).

## Methods

Since October 2021, the Work Group has conducted a systematic literature search and reviewed available evidence regarding the efficacy and safety of nirsevimab (*2*,*3*). The Work Group considered a priori outcomes that were critical or important to policy decisions.^§^ For infants born during or entering their first RSV season, evidence regarding efficacy and safety was derived from multicountry trials^¶^ that randomized infants, in a 2:1 ratio, to receive nirsevimab or placebo; a phase 2b trial that enrolled 1,453 preterm infants born at 29–34 weeks’ gestation (phase 2b trial) (*17*); and a phase 3 trial that enrolled 3,012 late preterm and term infants born at ≥35 weeks’ gestation (phase 3 trial) (*18*).** For children at increased risk for severe disease entering their second RSV season, evidence regarding efficacy and safety was obtained from a multicountry trial that randomized children to receive nirsevimab or palivizumab (*19*). The Work Group used the GRADE approach to assess the certainty of evidence for outcomes related to nirsevimab, rated on a scale of very low to high certainty (*2*,*3*). The Work Group then used the EtR Framework to guide its deliberations on recommendation of nirsevimab, reviewing data on the public health problem, benefits and harms, value to the target population, acceptability to key stakeholders, feasibility, resource use, and equity (*4*,*5*).

## Nirsevimab Efficacy and Safety

Among infants aged <8 months who were born during or entering their first RSV season, efficacy was evaluated through 150 days after injection. For the GRADE assessment, results from the phase 3 and phase 2b trials were pooled (*17*,*18*). Only infants who received the recommended dose of nirsevimab were included in pooled estimates.^††^ Pooled efficacy in preventing medically attended RSV-associated LRTI^§§^ was 79.0% (95% CI = 68.5%–86.1%; 31 of 2,579 in nirsevimab arm and 80 of 1,293 in placebo arm), efficacy in preventing RSV-associated LRTI with hospitalization was 80.6% (95% CI = 62.3%–90.1%; 12 of 2,579 in nirsevimab arm and 33 of 1,293 in placebo arm), and efficacy in preventing RSV-associated LRTI with ICU admission was 90.0% (95% CI = 16.4%–98.8%; one of 2,579 in nirsevimab arm and six of 1,293 in placebo arm). No deaths attributable to RSV were reported in either trial.^¶¶^ The incidence of serious adverse events*** was not increased in the nirsevimab arm compared with that in the placebo arm.^†††^ The overall evidence certainty using GRADE criteria was rated as moderate. The GRADE evidence profile and supporting evidence for the EtR Framework are available at https://www.cdc.gov/vaccines/acip/recs/grade/nirsevimab-season1-rsv-infants-children.html and https://www.cdc.gov/vaccines/acip/recs/grade/nirsevimab-season1-rsv-infants-children-etr.html.

Among infants at increased risk for severe disease who are entering their second RSV season, evidence was derived from a single trial that enrolled 615 preterm infants born at <35 weeks’ gestation who were eligible to receive palivizumab and 310 infants with either chronic lung disease requiring medical intervention within 6 months of randomization or hemodynamically significant congenital heart disease (CHD) (*19*). Participants were randomized to receive nirsevimab or palivizumab.^§§§^ Efficacy against medically attended RSV-associated LRTI was extrapolated from pharmacokinetic data.^¶¶¶^ Nirsevimab concentration levels among infants and children aged ≤24 months with chronic lung disease or CHD who received 200 mg nirsevimab entering their second RSV season were comparable to levels among those who received 50 mg if weighing <5 kg (<11 lb) and 100 mg if weighing ≥5 kg (≥11 lb) in their first RSV season. During the participants’ second RSV season, the incidence of serious adverse events did not significantly differ between the nirsevimab and palivizumab arms. The overall evidence certainty using GRADE criteria was rated as very low. Because nirsevimab appears to have efficacy as high as, or higher than, palivizumab (although no head-to-head efficacy trials exist) (*20*), and is assumed to be less costly (*21*), replacing palivizumab with nirsevimab for the palivizumab-eligible children entering their second season is expected to be cost saving. The GRADE evidence profile and supporting evidence for the EtR Framework are available at https://www.cdc.gov/vaccines/acip/recs/grade/nirsevimab-season2-rsv-infants-children.html and https://www.cdc.gov/vaccines/acip/recs/grade/nirsevimab-season2-rsv-infants-children-etr.html.

## Cost Effectiveness

The cost effectiveness for use of nirsevimab for infants aged <8 months born during or entering their first RSV season (at $445 per dose) was estimated to be $102,811 per quality adjusted life year (*21*). Because infants and children entering their second RSV season are at reduced risk for severe RSV disease compared with infants during their first RSV season, cost effectiveness for use of nirsevimab for the general population of children entering their second season (at $890 per dose)**** was estimated to be $1,557,544 per quality adjusted life year (*21*). Data to assess the incidence of severe RSV disease and death by type of chronic disease during their second RSV season are limited (*21*), as are data on efficacy and safety of nirsevimab among infants and children in their second RSV season.

## Recommendations for Use of Nirsevimab

ACIP recommends 1 dose of nirsevimab for all infants aged <8 months born during or entering their first RSV season (50 mg for infants weighing <5 kg [<11 lb] and 100 mg for infants weighing ≥5 kg [≥11 lb]). ACIP recommends 1 dose of nirsevimab (200 mg, administered as two 100 mg injections given at the same time at different injection sites) for infants and children aged 8–19 months who are at increased risk for severe RSV disease and entering their second RSV season^††††^ (Box). The recommendations for nirsevimab apply to infants and children recommended to receive palivizumab by AAP.^§§§§^ These recommendations will be updated as new evidence becomes available.

BOX **Infants and children aged 8–19 months with increased risk for severe disease who are recommended to receive nirsevimab when entering their second respiratory syncytial virus season**

- Children with chronic lung disease of prematurity who required medical support (chronic corticosteroid therapy, diuretic therapy, or supplemental oxygen) any time during the 6-month period before the start of the second RSV season
- Children with severe immunocompromise
- Children with cystic fibrosis who have either 1) manifestations of severe lung disease (previous hospitalization for pulmonary exacerbation in the first year of life or abnormalities on chest imaging that persist when stable), or 2) weight-for-length <10th percentile
- American Indian or Alaska Native children

**Abbreviation:** RSV = respiratory syncytial virus.

## Clinical Guidance

### Timing of Nirsevimab Administration

Providers should administer nirsevimab to infants aged <8 months and to infants and children aged 8–19 months who are at increased risk for severe RSV disease beginning shortly before the start of the RSV season. On the basis of pre–COVID-19 pandemic patterns, nirsevimab could be administered in most of the continental United States from October through the end of March. Infants born shortly before or during the RSV season should receive nirsevimab within 1 week of birth. Nirsevimab administration can occur during the birth hospitalization or in the outpatient setting. Optimal timing for nirsevimab administration is shortly before the RSV season begins; however, nirsevimab may be administered to age-eligible infants and children who have not yet received a dose at any time during the season. Only a single dose of nirsevimab is recommended for an RSV season. Infants with prolonged birth hospitalizations related to prematurity or other causes should receive nirsevimab shortly before or promptly after hospital discharge.^¶¶¶¶^ No evidence is available to support use of nirsevimab for prevention of hospital-acquired RSV infection, and nirsevimab is not recommended for this indication.

Because the timing of the onset, peak, and decline of RSV activity might vary geographically, providers can adjust administration schedules based on local epidemiology. RSV seasonality in tropical climates (including southern Florida, Guam, Hawaii, Puerto Rico, U.S.-affiliated Pacific Islands, and U.S. Virgin Islands) might differ from that of most of the continental United States or be unpredictable (*21*–*23*). In Alaska, RSV seasonality is less predictable, and the duration of RSV activity is often longer than the national average duration (*24*). Providers in these jurisdictions should consult state, local, or territorial guidance on timing of nirsevimab administration.

### Coadministration with Routine Childhood Vaccines

On the basis of limited data from clinical trials, coadministration of nirsevimab with routine vaccines resulted in a similar rate of adverse events compared with administration of vaccines alone (*25*). Nirsevimab is not expected to interfere with the immune response to other routine childhood immunizations (*26*). In accordance with general best practices for immunization, simultaneous administration of nirsevimab with age-appropriate vaccines is recommended (*27*).

### Infants and Children Aged 8–19 Months at Increased Risk for Severe RSV Disease and Entering Their Second RSV Season

Infants and children aged 8–19 months who are at increased risk for severe RSV disease and who are entering their second RSV season (timing of season as defined above) are recommended to receive nirsevimab. Replacing palivizumab with nirsevimab is expected to be cost saving, and ACIP recommends nirsevimab for eligible children entering their second RSV season, similar to groups of children recommended by AAP for palivizumab during their second RSV season (*16*) (Box). In addition, research suggests that some American Indian or Alaska Native (AI/AN) children experience high rates of severe RSV disease. A recent study found that incidence of RSV-associated hospitalization among some AI/AN children aged 12–23 months was four to 10 times that of similar-aged children across seven sites in the United States (*28*). These studies have been limited to specific populations and might not be broadly representative of risk in all AI/AN children. Some AI/AN communities live in remote regions, making transportation of children with severe RSV more challenging (*16*). Given the available evidence, ACIP also recommends nirsevimab for AI/AN children entering their second RSV season.

### Precautions and Contraindications

When administering nirsevimab to children with increased risk for bleeding, providers should follow ACIP’s general best practice guidelines for immunization (*27*). Nirsevimab is contraindicated in persons with a history of severe allergic reaction (e.g., anaphylaxis) after a previous dose or to a product component. Adverse reactions might occur after administration of nirsevimab alone; these reactions may be reported to MedWatch online (https://www.fda.gov/medwatch), by fax, by mail, or by contacting FDA at 1-800-FDA-1088.*****

Adverse reactions might occur after the coadministration of nirsevimab with a vaccine; these reactions should be reported to the Vaccine Adverse Event Reporting System (VAERS), and reports should specify that the patient received nirsevimab on the VAERS form.^†††††^ Reports can be submitted to VAERS online, by fax, or by mail. Additional information about VAERS is available by telephone (1-800-822-7967) or online (https://vaers.hhs.gov). When adverse reactions that occur after the coadministration of nirsevimab with a vaccine are reported to VAERS, additional reporting of the same adverse reactions to MedWatch is not necessary.

## Footnotes

*
https://www.fda.gov/news-events/press-announcements/fda-approves-new-drug-prevent-rsv-babies-and-toddlers

† The recommended dosage for infants born during or entering their first RSV season and weighing <5 kg (<11 lb) is 50 mg; for those weighing ≥5 kg (≥11 lb), the recommended dosage is 100 mg. The recommended dosage for infants and children aged 8–19 months at increased risk for severe disease entering their second RSV season is 200 mg (2 x 100 mg injections).

§ Critical outcomes include medically attended RSV-associated LRTI, RSV-associated LRTI with hospitalization, RSV-associated LRTI with ICU admission, and RSV-associated death. Important outcomes include all-cause medically attended LRTI, all-cause LRTI-associated hospitalization, and serious adverse events.

¶ Phase 2b trial locations: Argentina, Australia, Brazil, Bulgaria, Canada, Chile, Czechia, France, Hungary, Italy, New Zealand, Poland, Russia, South Africa, Spain, United Kingdom, and United States; phase 3: Austria, Belgium, Bulgaria, Canada, Czechia, Estonia, Finland, France, Germany, Israel, Japan, Latvia, Lithuania, Poland, Russia, South Africa, South Korea, Spain, Sweden, United Kingdom, and United States.

** An additional trial was conducted that enrolled 615 preterm infants born at <35 weeks’ gestation and who were eligible to receive palivizumab and 310 infants with either chronic lung disease and requiring medical intervention within 6 months of randomization or hemodynamically significant CHD. Participants were randomized (2:1) to either receive 1 dose of nirsevimab or monthly injections of palivizumab. The trial was designed as a pharmacokinetic study and was not designed to measure efficacy. A nirsevimab concentration target was established based on the phase 2b and phase 3 trials. The preterm, CHD, and chronic lung disease cohorts all met the threshold. In addition, day 150 postinjection concentrations in the increased risk trial were comparable or higher than in the phase 3 trial. This study did not meet criteria for inclusion in GRADE for efficacy of infants in their first RSV season because there was no placebo control group.

†† In the phase 2b trial, all infants in the treatment arm received 50 mg nirsevimab. Among infants who weighed ≥5 kg (≥11 lb), nirsevimab concentrations and efficacy were found to be lower. In the phase 3 trial, the dose remained 50 mg for those who weighed <5 kg (<11 lb) and increased to 100 mg for those who weighed ≥5 kg (≥11 lb). Among 969 infants in the phase 2b trial treatment arm, 399 (41%) were excluded from pooled analyses.

§§ Medically attended LRTI was defined as at least one documented physical examination finding localized to the lower respiratory tract, clinical signs and symptoms of severe respiratory disease, an inpatient or outpatient encounter, and a positive RSV polymerase chain reaction test result.

¶¶ For benefit outcomes rated as important for policy decisions by the Work Group, nirsevimab lowered the risk for all-cause medically attended LRTI (efficacy = 34.8% [95% CI = 23.0%–44.7%]) and all-cause LRTI-associated hospitalization (efficacy = 44.9% [95% CI = 24.9%–59.6%]).

*** Serious adverse events were defined in the protocol as any adverse event that results in death, is immediately life-threatening, requires inpatient hospitalization or prolongs an existing hospitalization, results in persistent or significant disability/incapacity, or is an important medical event that might jeopardize the subject or might require medical intervention to prevent one of the outcomes listed.

††† No adverse events of anaphylaxis or immune complex disease were reported. Two adverse events of special interest, both thrombocytopenia, were reported. One event was diagnosed as heparin-induced thrombocytopenia, and one occurred in a patient with a diagnosis of sepsis; neither was assessed as being attributable to or related to nirsevimab. Among the initially enrolled 1,490 infants in the phase 3 trial, the incidence of medically attended RSV-associated LRTI 351–510 days after injection was not significantly different in the nirsevimab (0.7%) and control (0.2%) arms, suggesting that protection provided from nirsevimab does not result in a shift in the RSV burden to the second year of life. The incidence of new onset chronic disease was similar in the nirsevimab (0.3%) and placebo (0.4%) arms. Among all participants in the phase 2b and phase 3 trials, adverse events were reported in 1.2% of participants who received nirsevimab within 360 days of the injection. Most (97%) of these were mild to moderate in intensity. Adverse reactions that were more common among infants who received nirsevimab than placebo were rash occurring within 14 days of injection (0.9% of nirsevimab recipients versus 0.6% of placebo recipients) and injection site reactions occurring within 7 days of injection (0.3% of nirsevimab recipients versus 0% of placebo recipients).

§§§ Among infants in their first RSV season, those in the nirsevimab arm received 50 mg if they weighed <5 kg (<11 lb) and 100 mg if they weighed ≥5 kg (≥11 lb). Participants with chronic lung disease or CHD who received nirsevimab in season 1 also received nirsevimab in season 2, and those who received palivizumab in season 1 were rerandomized in a 1:1 ratio to receive nirsevimab or palivizumab in season 2. In season 2, nirsevimab was administered as a 200 mg dose followed by four monthly injections of placebo. Palivizumab was administered as 5 monthly 15 mg/kg doses.

¶¶¶ Pharmacokinetic extrapolation was used and based on comparable pharmacokinetic levels from efficacy data among infants aged <12 months for prevention of the first medically attended RSV-associated LRTI to pharmacokinetic levels among infants and children aged ≤24 months with chronic lung disease or CHD entering their second RSV season. On the basis of pharmacokinetic and efficacy data from the phase 2b and phase 3 (MELODY) trials, a target area under the curve nirsevimab concentration of >12.8 mg*day/mL was established. For the chronic lung disease cohort, 129 of 132 (98%) participants met the target nirsevimab concentration, and for the CHD cohort, all participants met the target. In addition, the concentration of nirsevimab 150 days after injection was higher compared with the 150-day concentration in the phase 3 trial nirsevimab arm population.

**** Assumes that the cost of 200 mg of nirsevimab will be twice that of 100 mg. The cost of 50 mg and 100 mg of nirsevimab was assumed to be the same. https://www.cdc.gov/vaccines/acip/meetings/downloads/slides-2023-08-3/02-RSV-jones-508.pdf

†††† Infants and children aged ≥8 months have likely experienced an RSV season and are at decreased risk for severe RSV-associated disease compared with younger infants without previous RSV exposure. Children aged ≥20 months have likely experienced two RSV seasons and are at decreased risk for severe disease compared with younger children who have experienced only one RSV season.

§§§§ AAP has released guidance on the use of palivizumab and nirsevimab. https://publications.aap.org/redbook/resources/25379

¶¶¶¶ Consistent with general best practices for immunization, the chronologic (not corrected) age of preterm infants should be used to determine timing and eligibility for nirsevimab administration.

***** Adverse events can be reported to MedWatch because FDA has classified nirsevimab as a drug.

††††† Specifically, in Section 9: “Prescriptions, over-the-counter medications, dietary supplements, or herbal remedies being taken at the time of vaccination.”
